# Focal axonal swellings and associated ultrastructural changes attenuate conduction velocity in central nervous system axons: a computer modeling study

**DOI:** 10.1002/phy2.59

**Published:** 2013-08-28

**Authors:** Katarina V Kolaric, Gemma Thomson, Julia M Edgar, Angus M Brown

**Affiliations:** 1 School of Biomedical Sciences, Queens Medical Centre, University of NottinghamNottingham, NG7 2UH, U.K; 2 Institute of Infection, Immunity and Inflammation, College of Medical, Veterinary and Life Sciences, University of GlasgowGlasgow, G61 1QH, U.K; 3 Department of Neurology, University of WashingtonSeattle, Washington, 98195

**Keywords:** Aglycemia, axonal spheroid, myelin, permeability

## Abstract

The constancy of action potential conduction in the central nervous system (CNS) relies on uniform axon diameter coupled with fidelity of the overlying myelin providing high-resistance, low capacitance insulation. Whereas the effects of demyelination on conduction have been extensively studied/modeled, equivalent studies on the repercussions for conduction of axon swelling, a common early pathological feature of (potentially reversible) axonal injury, are lacking. The recent description of experimentally acquired morphological and electrical properties of small CNS axons and oligodendrocytes prompted us to incorporate these data into a computer model, with the aim of simulating the effects of focal axon swelling on action potential conduction. A single swelling on an otherwise intact axon, as occurs in optic nerve axons of *Cnp1* null mice caused a small decrease in conduction velocity. The presence of single swellings on multiple contiguous internodal regions (INR), as likely occurs in advanced disease, caused qualitatively similar results, except the dimensions of the swellings required to produce equivalent attenuation of conduction were significantly decreased. Our simulations of the consequences of metabolic insult to axons, namely, the appearance of multiple swollen regions, accompanied by perturbation of overlying myelin and increased axolemmal permeability, contained within a single INR, revealed that conduction block occurred when the dimensions of the simulated swellings were within the limits of those measured experimentally, suggesting that multiple swellings on a single axon could contribute to axonal dysfunction, and that increased axolemmal permeability is the decisive factor that promotes conduction block.

## Introduction

One of the key pathologies associated with central white matter axons is disruption of the myelin sheath that surrounds axons. This can manifest either as demyelination, where existing myelin breaks down, or dysmyelination, where myelin formation is abnormally affected. Demyelination is a hallmark of multiple sclerosis (MS), an inflammatory condition resulting in focal white and gray matter lesions. Other myelin pathologies affecting the central nervous system (CNS) include genetic abnormalities resulting in more generalized hypomyelination such as the leukodystrophies (Quarles et al. 2006) and developmental pathologies such as periventricular leukomalacia (Folkerth 2006). The effects of demyelination have been extensively studied from the cellular level to the whole animal and may be summarized, simply, as follows: demyelination leads to attenuated conduction velocity or conduction block, with chronic demyelination possibly leading to the death of underlying axons, resulting in irreversible loss of function. Secondary, focal demyelination can also result from swelling of the axon underlying the myelin, where the physical expansion of the axon disrupts the integrity of the myelin sheath (Edgar et al. 2004), or as a consequence of axonal demise.

Previous studies have shown focal axonal swelling as a result of expression of the null mutation of genes encoding myelin proteolipid (*PLP1/Plp1*) or 2′,3′-cyclic nucleotide 3′-phosphodiesterase (*Cnp1*) (Griffiths et al. 1998; Garbern et al. 2002; Edgar et al. 2004), or energy insufficiency resulting from aglycemia (Allen et al. 2006), anoxia (Waxman et al. 1992), or ischemia (Garthwaite et al. 1999). In *Plp1* and *Cnp1* null mouse mutants, the axon swellings occur in conjunction with accumulation of intraaxonal organelles such as mitochondria and dense bodies (Griffiths et al. 1998), reflecting impaired axonal transport (Edgar et al. 2004). In the *Plp1* knockout mouse, axonal swellings tend to be located at the distal juxtaparanodal region of the axon. These swellings are up to ∼10 μm in length with an equivalent diameter, and a frequent feature is the eventual retraction and attenuation of the myelin sheath (Edgar et al. 2004). An additional feature of at least some pathological axonal swellings is increased axolemmal permeability (Pettus and Povlishock 1996; Povlishock and Pettus 1996; Fitzpatrick et al. 1998; Choo et al. 2008), as demonstrated by transmembrane movement of normally excluded horseradish peroxidase. The increased permeability of the axolemma would act as a shunt to dissipate axoplasmic current across the axolemma. Given the intimate relationship between axon structure and conduction, and the clear disruption to axon structure resulting from focal swelling of axons, one would reasonably expect axonal swellings to disrupt axon conduction, but the relationship between attenuation of conduction velocity and magnitude of swelling remains unclear.

In this study we assimilated recently published data describing morphological and electrical properties derived from imaging and electrophysiological experiments on CNS white matter axons and oligodendrocytes (Bakiri et al. 2011), with the aim of developing a computer model representative of small (<0.5 μm diameter) CNS axons, in order to examine the effects of simulated axon swellings on conduction velocity. The model incorporated morphological and electrical parameters from corpus callosum axons and oligodendrocytes (Bakiri et al. 2011), and voltage-gated conductances from historical data (Richardson et al. 2000) at the nodes. As a first step, the effects of demyelination on conduction velocity were investigated, followed by the effects of populating demyelinated axons with two types of Na^+^ channel that are expressed at normal axons/nodes. Subsequently, simulations of a single-axon swelling at the distal juxtaparanodal region of the internodal region (INR), and multiple swellings in the INR were modeled. Our results demonstrate that single swellings accompanied by demyelination of the swollen region attenuate conduction, whereas multiple swellings in similarly demyelinated axons demonstrated greater attenuation of conduction for equivalent dimensions of single swellings. Increasing axolemmal permeability greatly decreased the dimensions of a swelling that resulted in conduction block; dimensions that were within the range of those measured experimentally, suggesting that the most important factor that leads to conduction block in swollen axons is not the swelling or accompanying demyelination, but the increased axonal permeability, which acts as a transmembrane current shunt.

## Methods

All procedures were carried out in accordance with the Animals (Scientific Procedures) Act 1986 under appropriate authority of project and personal licenses.

### Electrophysiology

Recordings were made from *Cnp1* null mice and wild-type (WT) littermates (Lappe-Siefke et al. 2003; Edgar et al. 2009), or CD-1 mice. Mice were killed by cervical dislocation and optic nerves dissected free and cut behind the orbit and at the chiasm. The nerves were placed in an interface perfusion chamber (Medical Systems Corp, Greenvale, NY), maintained at 37°C, and superfused with artificial cerebrospinal fluid (aCSF) containing (in mmol/L): NaCl 126, KCl 3.0, CaCl_2_ 2.0, MgCl_2_ 2.0, NaH_2_PO_4_ 1.2, NaHCO_3_ 26, and glucose 10. The chamber was continuously aerated by a humidified gas mixture of 95% O_2_/5% CO_2_. Nerves were allowed to equilibrate in standard aCSF for about 30 min before beginning an experiment. Suction electrodes back filled with aCSF were used for stimulation and recording. During an experiment, the compound action potential (CAP) was elicited every 30 sec. The signal was amplified 1000× by a Stanford Research Systems Preamplifier (SR560, Stanford Research Systems, Sunnyvale, CA), filtered at 10 kHz, and acquired at 20 kHz (Clampex 9.2, Molecular Devices, Wokingham, U.K.).

### Transmission electron microscopy

Adult male CD-1 mice (35–45 g: 56 days and older) were obtained from Charles Rivers, U.K. The mice were killed by cervical dislocation and then decapitated. Optic nerves were dissected free and cut at the optic chiasm and behind the orbit. Mouse optic nerves (MONs) were laid out on cardboard and fixed in 2% glutaraldehyde and 2% paraformaldehyde solution in 0.2-M phosphate buffer overnight and postfixed in 1% osmium tetroxide for 30 min as previously described (Allen et al. 2006). They were dehydrated in a graded ethanol series and embedded in transmit low viscosity resin (TAAB Laboratories Equipment Ltd., Aldermaston, U.K.). Ultrathin sections (70–90 nm) were prepared using a Reichert-Jung Ultracut E ultramicrotome and mounted on 100 hexagonal copper grids. They were contrasted using uranyl acetate and lead citrate and viewed using a JEOL 1010 Transmission Electron Microscopy (TEM) operated at 80 kV with digital image acquisition.

### Transgenic animals and genotyping

All animal studies were approved by the Ethical Committee of the University of Glasgow and licensed by the U.K. Home Office. *Cnp1* knockout mice (Lappe-Siefke et al. 2003), backcrossed for six generations to the C57BL6N (Charles River) background, were crossed to the *Thy1-cyan fluorescent protein* (*CFP*) line (Feng et al. 2000) (B6.Cg-Tg(Thy1-CFP)23Jrs/J) to generate *Cnp1* homozygous knockout*Thy1-CFP hemizygous offspring.

Genomic DNA was isolated from tail biopsies using a DNA isolation kit (Promega Corporation, Madison, WI), and polymerase chain-reaction (PCR) reactions were performed using RedTaq (Sigma-Aldrich, Poole, Dorset, U.K.). Primers for identification of *Cnp1* alleles were described previously (Lappe-Siefke et al. 2003). *Thy1-CFP* hemizygous mice were identified by the presence of CFP-positive axons in ear clippings. Ear clippings were collected freshly, split to expose the dermis, mounted on a microscope slide, and viewed under an Olympus IX70 microscope using filters for fluorescein isothyocyanite (FITC).

### Immunohistochemistry

*Cnp1* knockout*Thy1-CFP mice were perfusion fixed with physiological saline followed by 4% paraformaldehyde. The optic nerves were dissected and cryoprotected in 20% sucrose, then rapidly frozen in Tissue Tec OCT embedding medium (Fintek Europe, Zoeterwoude, The Netherlands) in liquid nitrogen chilled isopentane. Ten-micron-thick longitudinal sections were cut on a crytostat (Bright Instrument Co. Ltd., Huntingdon, U.K.). CFP labeling was enhanced using immunohistochemical staining with a primary antibody to GFP (ab 6446, Abcam, Cambridge, U.K.; 1:1000), which was visualized with a goat anti-rabbit FITC-labeled secondary antibody (Cambridge Biosciences, U.K.).

### Computer simulations

**Figure 1:**
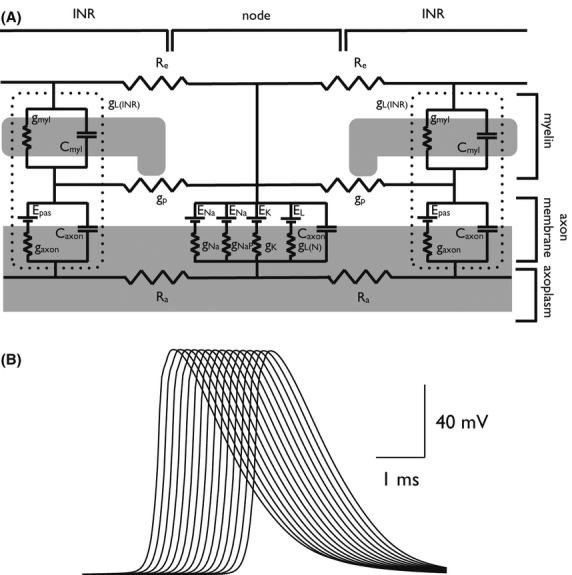
Equivalent circuit of the model corpus callosum axon, which has been adapted from existing models (Richardson et al. 2000; Devaux and Gow 2008). (A) The axon is divided into two regions, the node and internodal (INR). The nodal region expresses voltage-dependent conductances, as well as leak current (g_L_) and capacitance (C_axon_). The axolemma underlying the myelin also has these properties (g_axon_ and C_axon_), but the myelin contributes an additional resistive (g_myl_) and capacitative barrier (C_myl_). The dotted line encloses the components comprising leak and capacitance of the INR. The passive current across the membrane (g_L_) is the sum of g_myl_ and g_axon_. Axon resistance (R_a_) is constant throughout the model. Although not included in this model, claudin 11 forms tight junctions between the paranodal loops of myelin and the axon presenting a resistive barrier between the extracellular fluid and periaxonal space (g_p_) (Devaux and Gow 2008). External resistance (R_e_) is 0. (B) Action potentials are recorded at sequential nodes (6–20).

The simulations were carried out using NEURON 7.1 (Hines and Carnevale 1997). The cell builder module was used to construct a model consisting of a soma from which emerged a single axon. The axon comprised alternating myelinated internodal regions (INR) and unmyelinated nodal compartments, with 26 of the former and 25 of the latter. The morphological dimensions, and passive measures of conductance, resistance, and capacitance were based on averaged data from multiple corpus callosum axons and oligodendrocytes (Bakiri et al. 2011), and were integrated to construct the basic axon model. The voltage-dependent conductances (Richardson et al. 2000) upon which action potential conduction depends were incorporated into the nodal compartments (Fig. 1A). Simulated action potentials were computed using backward Euler integration with a time step of 0.01 msec. Conduction velocity was calculated based on action potential propagation between nodes 6 and 20. Any changes in individual INR lengths were taken into account in the calculations.

### Passive membrane properties

The basic passive electrical properties for the INRs of the axon are summarized in Table 1, where R_a_ is the axoplasmic resistance, C_m_ is the combined capacitance of the axolemma (C_axon_) and myelin (C_myl_), g_L_ is the combined conductance of the axolemma and myelin at the INR, and the conductance of the axolemma at the node, and e_L_ is the reversal potential for I_L_. The soma measured 20 μm by 20 μm with R_a_ = 70 Ωcm, C_m_ = 0.9 μF cm^−2^, and I_Na_, I_K_, and I_NaP_ as described in the following section. The soma was the site of current injection. The INRs were 79.1 μm in length and 0.36 μm in diameter, whereas the nodal regions were 1 μm in length and 0.36 μm in diameter. The axon membrane was modeled with nodal g_L_ = 80 mS cm^−2^ and INR g_L_ = 0.1 mS cm^−2^, C_m_ = 0.9 μF cm^−2^, E_L_ = −83.4 mV, and the axoplasmic resistance R_a_ = 70 Ω cm. Each myelin membrane was modeled with g_L_ = 0.553 mS cm^−2^, C_m_ = 0.9 μF cm^−2^, and a thickness of 0.012 μm. As each wrap of myelin consisted of two myelin lamella, the number of lamella was doubled. For each INR the total conductance was calculated as the axolemma in series with the appropriate number of myelin lamella such that:


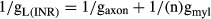
(1)

where *n* is the number of myelin wraps. Similarly, the INR capacitance was calculated based on the axolemma in series with myelin, thus:


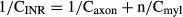
(2)

where *n* is the number of myelin wraps. The length constant (λ) was calculated as:


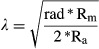
(3)

**Table 1:** Morphological and passive electrical properties of the axonal and nodal compartments

	0	1	2	3	4	5	Node
L (μm)	79.1	79.1	79.1	79.1	79.1	79.1	1
D (μm)	0.36	0.384	0.408	0.432	0.458	0.48	0.36
R_a_ (Ω cm)	70	70	70	70	70	70	70
C_m_ (μF cm^−2^)	0.9	0.3	0.18	0.1286	0.1	0.0818	0.9
g_L_ (mS cm^−2^)	0.1	0.0734	0.058	0.048	0.0409	0.0356	80
e_L_ (mV)	−83.4	−83.4	−83.4	−83.4	−83.4	−83.4	−83.4
λ (μm)	358.5	418.4	470.7	517.7	560.9	600.9	

The numbers heading of the columns indicate the number of myelin wraps, with the values of D, C_m_, g_L_, and λ altered accordingly. The rightmost columns contain the nodal properties

where R_m_ is the membrane resistance (= 1/g_L(NR)_), R_a_ is the axon resistance, and rad is the axon radius (Nicholls et al. 2001). These passive parameters for axons ensheathed with up to five wraps of myelin, and equivalent values at the node are shown in Table 1.

Each INR was divided into compartments such that the length of each compartment was less than 0.1 λ to ensure each compartment is isopotential, the generally accepted practice in such simulations (Carnevale and Hones 2006; Sterratt et al. 2011).

### Voltage-dependent conductances

The voltage-dependent conductances incorporated into the model were a fast sodium current (I_Na_), a persistent sodium current (I_NaP_), and a slow potassium conductance (I_K_), which were based on existing data (Richardson et al. 2000). The voltage-dependent conductances were g_Na_ = 3 S cm^−2^, g_NaP_ = 5 mS cm^−2^, and g_K_ = 80 mS cm^−2^. The values for their rate constants (RC) at 37°C have been computed from original data assuming *Q*_10_ values of 2.2 for I_Na_ and 3.0 I_K_ for I_NaP_, respectively (Richardson et al. 2000), and have been rearranged into a format compatible with the default equation input into NEURON. All RC were expressed in the form:


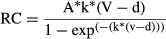
(4)

except for βh which was computed as:


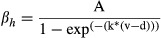
(5)

The parameter values are presented in Table 2.

**Table 2:** Rate constants (RC) parameters values of the voltage-dependent conductances

	A	k	d
αm	73.15	0.10	−25.41
βm	3.01	−0.11	−29.70
αh	1.42	−0.09	−118.11
βh	8.78	−0.075	−35.80
αn	0.186	0.042	−19.522
βn	0.133	−0.043	−97.99
αm_p_	7.319	0.097	−48.4
βm_p_	0.3	−0.11	−42.7

The RC are calculated with equation 4 except for βh, which is calculated with equation 5.

## Results

### Action potential conduction

Incorporating the voltage-dependent conductances at the nodes in combination with fully myelinating the axon allows simulation of propagated action potential conduction. The simulations illustrate the action potential profile, simulated inclusively from nodes 6 to 20 (Fig. 1B), in agreement with previously published models with regard to duration and amplitude (Richardson et al. 2000; Bakiri et al. 2011). The conduction velocity was calculated as 2.314 msec^−1^.

### Effect of demyelination on axon conduction

**Figure 2:**
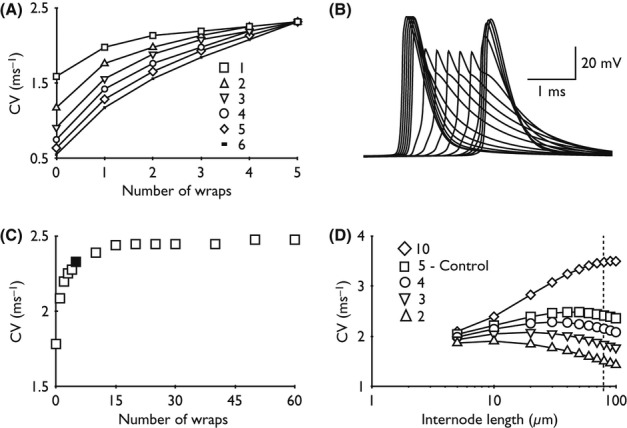
Effect of demyelination on conduction velocity. (A) The relationship between demyelination of multiple INRs on conduction velocity, where conduction velocity is calculated from the time difference between the peaks of action potentials recorded at nodes 6 and 16, is shown. The number of contiguous demyelinated INRs is indicated in the legend, and the number of wraps in each demyelinated region is indicated on the *x*-axis. (B) Action potentials recorded from nodes 6 to 20, where the INRs between nodes 12 and 16 were completely demyelinated, show that although demyelination does not result in conduction block, it does significantly slow conduction velocity. (C) Increasing the number of myelin wraps at each INR does not significantly increase conduction velocity. The control value of five wraps is indicated by the filled square. (D) Remyelination of INRs between nodes 11 and 20 with the number of wraps of myelin indicated in the legend shows that the shorter the INR the faster the conduction velocity. In control conditions, very small INR lengths results in a decrease in conduction velocity, with optimal conduction velocity occurring at about 50 μm. The dotted line indicates the control INR length of 79.1 μm.

In light of the focal demyelination that often accompanies axon swelling, we decided it was first necessary to investigate the effect of demyelination on conduction in our model. A valuable opportunity that computer simulations offer over laboratory-based experiments is the ability to selectively demyelinate both by degree and extent, that is, select the number of myelin wraps present and the number of INRs affected. We found that demyelination resulted in decreased conduction velocity, the greater the degree of demyelination, and the greater the number of INRs affected, the greater the attenuation of conduction velocity (Fig. 2A). Decreasing the degree of myelination incrementally resulted in sequential decreases in conduction velocity that became more profound the greater the degree of demyelination, that is, the slope of the relationship between number of myelin wraps and conduction velocity increased with increasing demyelination. This relationship held as the extent, that is, number of affected INRs, increased. However, even with complete demyelination at six INRs, conduction was not blocked, and indeed had fallen to about a third of the control value (Fig. 2A and B). It is interesting to note that the axon conducts at roughly the same conduction velocity when two INRs are completely demyelinated, as when six INRs possess only one wrap of myelin; that is, maintaining one wrap of myelin increases threefold the length of axon that can be demyelinated without sacrificing conduction velocity compared to a totally demyelinated axon (Fig. 2A). It appears that five wraps of myelin offer an optimal balance between increased conduction velocity relative to the increased volume occupied by myelin (Fig. 2C). The CNS is capable of remyelination after demyelination; thus, we investigated the relationship between the degree of remyelination and conduction velocity. Recent reports have measured an increase in both the g ratio (the ratio of the axonal diameter relative to the diameter of the fiber [axon plus its myelin sheath]), indicating the presence of fewer myelin wraps compared to control, and decreased INR length, following remyelination (Jarjour et al. 2012). We applied these data to simulations, where we decreased the number of myelin wraps from 5 to 2, and also increased the number of wraps to 10, and measured the resulting conduction velocity at various INR lengths, demonstrating that, in control myelinated axons, the conduction velocity increases and peaks when the INR length is about 50 μm, then slowly decreases to about 2.3 msec^−1^ at the control INR length of 79.1 μm. As expected, decreased myelin wraps and increased INR length conspired to attenuate conduction velocity, thus decreasing INR length after demyelination can be viewed as a protective means of preserving conduction velocity in the face of decreased myelination (Fig. 2D). The rise in conduction velocity as INR length increases followed by a fall at larger INRs can be explained as a balance between the INR length and time taken to charge the nodal membrane. At small INR lengths there is less distance for the intraaxonal current to travel between nodes, which would speed up conduction velocity, but this would be balanced by the increased number of nodes along the length of the axon requiring additional charging time for membrane capacitance.

### The effect of g_Na(P)_ expression on conduction velocity

**Figure 3:**
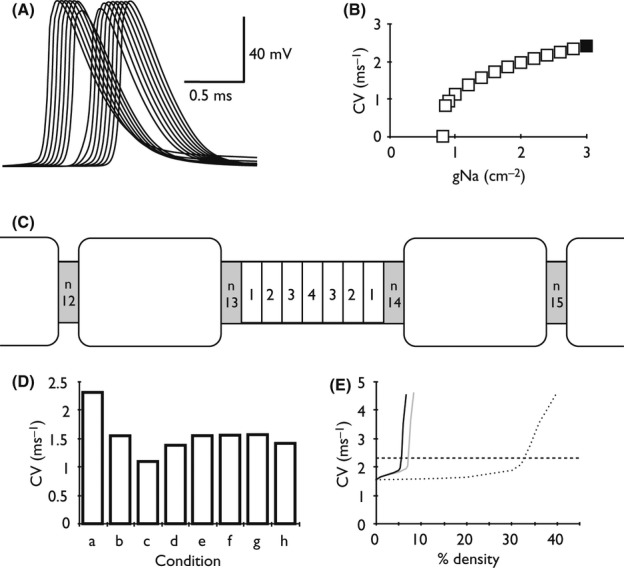
Na^+^ channel expression and conduction velocity. (A) Action potentials recorded at sequential nodes (6–20) with g_Na_ reduced to 1.0 S cm^−2^ at nodes 12 and 13. (B) Plot of conduction velocity versus nodal g_Na_ where the control g_Na_ value of 3 S cm^−2^ is indicated by the filled square. (C) Illustration of regions comprising the demyelinated INR. (D) Histogram of the conduction velocity resulting from the various distributions of I_Na_ and I_NaP_ described in the Results section. (E) Plot of density of g_Na_ (gray line), g_Na_ and g_NaP_ (bold line), and g_NaP_ (dotted line) relative to control nodal values versus conduction velocity.

The demyelinating model we describe in Figure 2 offers an ideal opportunity to model the changes in Na^+^ channel density that have been reported to occur after loss of myelin. The key aspect of these simulations was to determine whether redistribution of existing nodal Na^+^ channels onto the denuded axon could restore conduction velocity to control levels, or whether additional Na^+^ channels would be required, the latter indicative of generation of new Na^+^ channels proteins in the soma followed by trafficking down the axon. The safety factor for nodal g_Na_ was modeled by decreasing its value incrementally from 3 S cm^−2^ until conduction failed at 0.81 S cm^−2^, a safety factor of (3/0.81) 3.70 (Fig. 3A and B). There is evidence that after demyelination Na^+^ channel expression can alter such that nodal expression of Na^+^ channels decreases, but expression of Na^+^ channels on the bare axon increases (Craner et al. 2004). We modeled this phenomenon in the following way. An INR was modeled as demyelinated and divided into seven regions, of which there were three pairs of identical regions and a sole central region. Regions 1 abutted the nodes with Regions 2 adjacent to Regions 1; Regions 3 border Region 4, which lies in the center of the INR (Fig. 3C). Regions 1–3 were 10 μm long and Region 4 was 19.1 μm long. In these simulations our goal was to study the effect of redistribution of existing nodal Na^+^ channels on the bare axon on conduction velocity, to model the reported alterations in expression of the transient Nav1.2 channel and the persistent Nav1.6 channel (Craner et al. 2004; Waxman 2006). This was carried out in the following sequence, and the results are displayed in Figure 3D: (a) a control, myelinated nerve was modeled to provide a control conduction velocity for comparison; (b) the INR was unmyelinated with control values for nodal g_Na_ and g_NaP_ maintained; (c) the unmyelinated INR with no expression of g_Na_ or g_NaP_ at the nodes bordering the unmyelinated INR, that is, nodes 13 and 14; (d) the unmyelinated INR with only half the control values of nodal g_Na_ and g_NaP_; and (e) the unmyelinated INR with half control values of g_Na_ and g_NaP_ at the nodes, plus Regions 1 expressed g_Na_ of 0.15 S cm^−2^ and g_NaP_ of 0.005 S cm^−2^. As the surface area of Region 1 is 10 times that of the node, it was populated with a tenth the density of the node, ensuring that all the channels present at a control node are included, with half expressed at the node, and the remaining half expressed at the appropriate density on Region 1; (f) similar to (e) with half control values of g_Na_ and g_NaP_ at the nodes, but the remaining channels expressed on Regions 1 and 2 to simulate movement of channels from node to demyelinated axon; (g) similar expression of g_Na_ and g_NaP_ to (f) differing in that nodal expression of g_Na_ and g_NaP_ was one third that of control, and expression of g_Na_ was 0.1 S cm^−2^ on Regions 1 and 2, and g_NaP_ was 0.002 S cm^−2^ on Region 1 and 0.001 on Region 2; and (h) an even distribution of g_Na_ at the nodes and on Regions 1–4, which was 0.037 S cm^−2^ for g_Na_ and 0.0000616 S cm^−2^ for g_NaP_.

In the next simulation investigating the effect of Na^+^ channel density on conduction velocity, we maintained control nodal values of g_Na_ and g_NaP_, but populated the demyelinated INR with an even distribution of g_Na_ and/or g_NaP_ to determine what density of channel expression relative to that present at the node would be required to achieve the control conduction velocity. We did this in three stages, populating the INR with g_Na_ alone, g_Na_ plus g_NaP_, and finally g_NaP_ alone. We found that populating the INR with a density of g_Na_ and g_NaP_ of about 5.5% of their control values restored conduction velocity to control levels, with further marginal increases in conductance resulting in greatly increased conduction velocity. A similar effect was seen for g_Na_ alone with a density of about 7.2% of control restoring control conduction velocity. However, with g_NaP_ alone, a much higher expression of channels must be present (>30% of control, Fig. 3E).

### Repetitive firing

**Figure 4:**
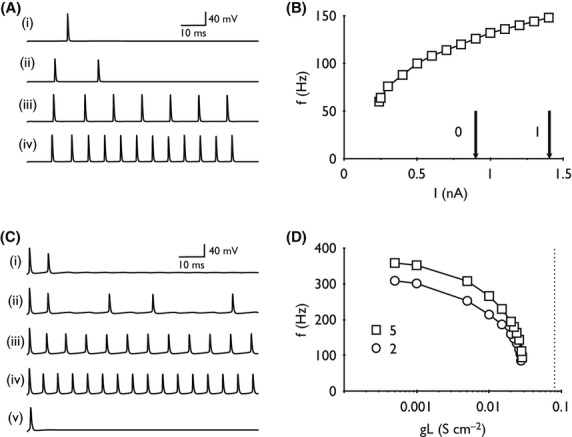
Repetitive firing. (A) Injecting current of increasing magnitudes induces: (i) an individual action potential (0.1 nA), (ii) two action potentials (0.23 nA) followed by (iii) repetitive firing (0.35 nA), whose frequency increases with (iv) increased stimulus current (1.3 nA). (B) The repetitive firing occurs over a limited range of frequencies. Decreasing the number of myelin wraps has minimal effect on the ability to fire repetitive action potentials, with only 0 wrap decreasing the upper limit to 120 Hz. Arrows indicate the position on the x-axis where repetitive fining fails with either 0 or 1 myelin wrap. (C) Decreasing the nodal g_L_ from the control value of 0.08 S cm^−2^ to 0.0287 S cm^−2^ induces action potentials in the absence of injected current (i), whose firing frequency increases with decreased g_L_ (0.0286 S cm^−2^), (ii) until spontaneous repetitive firing occurs (0.0280 S cm^−2^), (iii) whose frequency increases with decreased g_L_ (0.0250 S cm^−2^) (iv). Removal of g_NaP_ inhibits this repetitive firing (v). (D) The frequency range over which firing was supported extended from ∼100 to 380 Hz. Decreasing the number of myelin wraps from 5 to 2 decreased the firing frequency for all values of g_L_.

Existing models of the electrical properties of an axon are generally used to study aspects of action potential conduction, ranging from studying the ion currents underlying the action potential to the properties that govern repetitive firing. We wanted to demonstrate that our model, the first of small myelinated CNS axons, conforms to two recognized behaviors of similar models, namely, the effect of sustained current injection on repetitive firing (Koch 1999) and the effect of the nodal leak current (I_L_) on action potential activity (Coggan et al. 2010). Injection of current for an extended duration results in sustained repetitive firing in the model (Fig. 4A and B). However, below the threshold required to induce repetitive firing, current injections elicit a single action potential with further current injection eliciting further sporadic action potentials until repetitive firing occurs. The range over which this firing occurs is limited, between about 60 and 148 Hz, a phenomenon also reported in the Hodgkin Huxley squid giant axon model (Koch 1999). Demyelinating the axons only has an effect on the range of this *f–I* curve, when axons are totally denuded, limiting the upper range of firing to 120 Hz.

Repetitive firing can also be induced by reducing nodal g_L_, as previously reported in a model of myelinated central axons (Coggan et al. 2010) (Fig. 4C). Reducing g_L_ from the control value of 0.08 S cm^−2^ to 0.0287 S cm^−2^ results in a single action potential, whereas a further decrease to 0.0286 S cm^−2^ induces chaotic firing, that is, multiple action potentials unevenly spaced. At values of g_L_ lower than 0.0286 S cm^−2^ spontaneous repetitive firing occurs, with lower values of g_L_ resulting in increased firing rates. The range of frequencies at which firing is supported is wider than in response to injected current (Fig. 4B). Demyelinating the axon from 5 to 2 wraps of myelin reduces the firing frequency compared to fully myelinated axons over the entire range of g_L_ (Fig. 4D).

### Effect of a single-axon swelling on conduction velocity

**Figure 5:**
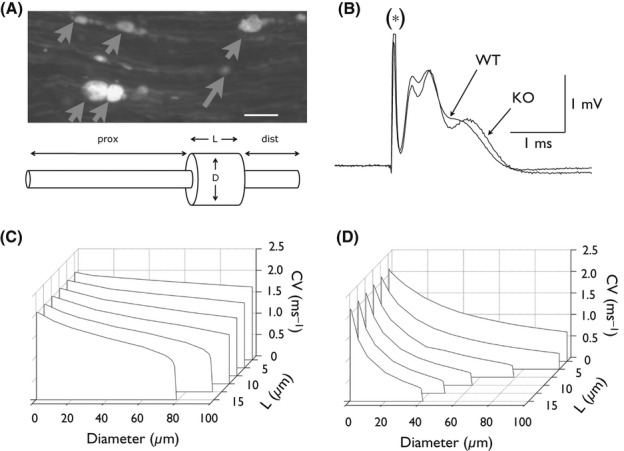
Effect of individual focal axonal swellings on conduction. A, upper. Ten-micrometer-thick section of the spinal cord of a *Cnp1*−/−*Thy1-CFP mouse. CFP labeling was enhanced using immunohistochemical staining with a primary antibody to GFP, which was visualized with a goat anti-rabbit FITC-labeled secondary antibody, to reveal focal swelling (arrows) of spinal cord axons. Scale bar 10 μm. A, lower. Schematic illustration of swollen region in distal section of axon. Length (L) and diameter (D) of the swollen region were altered in the model. (B) CAPs recorded from the optic nerves of wild-type (WT) and *Cnp1* null (KO) mice. *denotes the stimulus artifact. (C) Conduction velocity versus the length and diameter of a single swollen region illustrated in A, lower. As the length of the swollen region increases, the diameter at which conduction is blocked decreases. (D) Increasing the number of contiguous INRs with single swellings to eight significantly reduces the dimensions of each swollen region at which conduction is blocked.

In the *Cnp1* null mouse, the single axonal swellings that occur at the distal end of the INR remain myelinated (Edgar et al. 2009), affording the opportunity to investigate the effect of discrete axonal swelling on conduction velocity, by carrying out simulations based on our model, and also by carrying out electrophysiological recordings where we compared the CAP profile recorded from MONs from WT and *Cnp1* null mice. The CAP recorded from the MON is an extracellular recording, and the area under the CAP is the sum of the extracellular potentials recorded from all axons that fire action potentials in the response to the supramaximal stimulus. Thus, the area of the CAP is a monitor of all axons that contribute to the CAP with decreases in CAP indicative of action potential block. In *Plp1* null mice, axonal swellings frequently occur at the distal juxtaparanodal region (Griffiths et al. 1998). In *Cnp1* null mice, swellings can be observed also in INRs (Fig. 5A) (Edgar et al. 2009). CAPs recorded from WT and *Cnp1* null mice were indistinguishable in terms of latency to peak and CAP area (Fig. 5B). We simulated the effect of introducing a focally swollen region of axon of variable dimensions at the distal end of the INR by introducing a region of axon whose length and diameter we altered in size (Fig. 5A lower). The unaffected last portion of the INR (dist) was maintained at 10 μm in length, with increases in the length of the swollen region (L) compensated for by equivalent decreases in the proximal length of the INR (prox), such that the length of the affected INR was maintained at 79.1 μm. In these simulations, the swollen section of axon was modeled as myelinated as there was no evidence of widespread demyelination in these mice (Edgar et al. 2009). The action potentials were initiated at the soma and simulated action potentials were recorded at nodes 6–16 inclusively, with the INR between nodes 10 and 11 containing the focal axon swelling. Our simulations demonstrated only marginal attenuation of conduction velocity when the dimensions of the swollen region were within those seen in the axons of *Cnp1* null mice. However, for larger unphysiological dimensions, the decrease in conduction velocity was commensurate with the dimensions of the swollen region. Simulations were carried out where the length of the swollen region was increased in 2.5 μm steps up to 15 μm. We found that conduction block occurred only in swollen regions exceeding 12.5 μm in length and over 95 μm in diameter (Fig. 5C). It is likely, however, that such swellings occur over multiple INRs, thus we simulated single swellings over eight contiguous INRs (Fig. 5D). Qualitatively the results were similar to the simulations of a single swelling, but the dimensions at which conduction was blocked occurred at smaller swelling dimensions. For example, for a single swelling, conduction block occurred at a length of 15 μm for a diameter of 80 μm, but in multiple single swellings for an equivalent diameter the length was reduced to 7.5 μm. However, dimensions of >20 μm are clearly far larger than any swellings we have observed in our own studies, and are also incompatible with the reported literature (Adalbert et al. 2007), in agreement with the minimal effect on CAP profile seen in *Cnp1* null mice.

**Figure 6:**
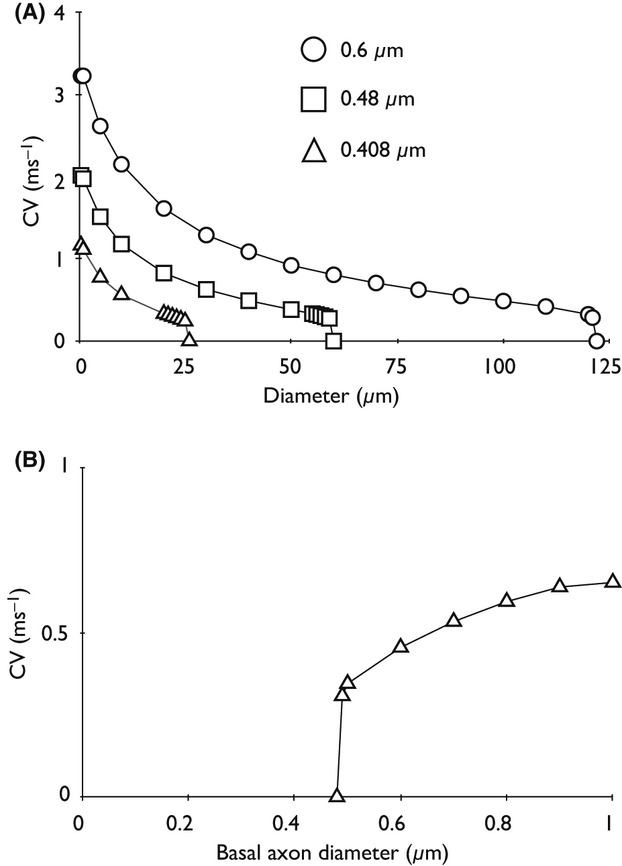
Effect of basal axon diameter on swelling-induced conduction block. (A) Simulating a single swollen region 10 μm in length in 8 contiguous INRs (as in Fig 5D) with basal axon diameters of 0.6 μm, 0.48 μm, or 0.408 μm resulted in increased conduction velocity with no swellings, as expected. Introduction of swellings caused conduction block at the lowest diameter for the thinnest axon (0.408 μm) and at the largest diameter for the thickest axon. (B) In a simulation using the same model as described in (A), conduction block occurs with swollen regions 10 μm in length and 60 μm in diameter. Maintaining the dimensions of the swollen regions, but increasing the basal diameter of the axon restores conduction in an incremental manner.

We investigated whether the base axon diameter is related to the minimum swelling diameter that causes conduction block. We repeated the simulation shown in Figure 5D with basal axon diameters of 0.6 μm, 0.48 μm (control), and 0.408 μm, with appropriate compensations made in C_m_ and g_L_ according to the values in Table 1. The length of the swellings was 7.5 μm and we varied the swelling diameter until conduction was blocked. The figure shows a clear relationship correlating smaller basal axon diameter and smaller diameter of swellings required to produce conduction block. In axons with a basal diameter of 0.408 μm conduction block occurred at 36 μm, 60-μm-diameter swellings were required to block conduction on axons with a basal diameter of 0.48 μm, and in the largest basal axon diameter we simulated (0.6 μm), conduction was blocked with swellings of 122 μm diameter (Fig. 6A).

The simulations illustrated in Figure 5D demonstrate that increasing the diameter of swellings causes a reduction in conduction velocity until a diameter is reached that is sufficiently large to block conduction. We next investigated the effect of maintaining the diameter of the swellings at the level that blocks conduction when axon diameter is of control value (0.48 μm), but increasing the basal axon diameter. We found that for swollen regions of 10 μm in length the diameter required to block conduction was 60 μm. Increasing the basal axon diameter from 0.48 μm where conduction is blocked, to 0.49 μm restored conduction, albeit at an attenuated velocity. Subsequent increases in axon diameter up to 1 μm resulted in incremental increases in conduction velocity (Fig. 6B)

### Effect of multiple axon swellings in a single INR on conduction velocity

**Figure 7:**
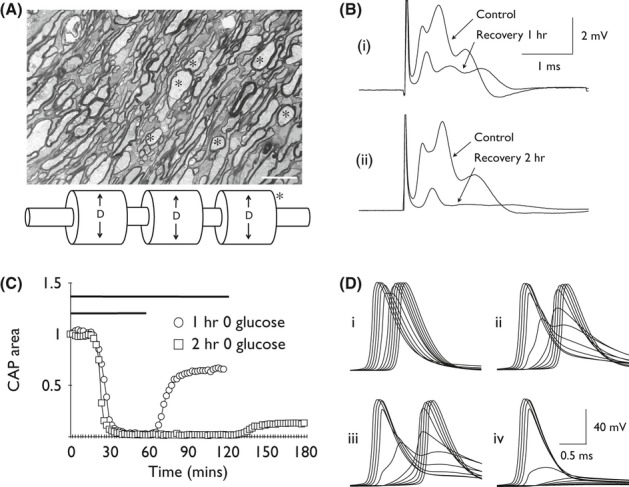
Effect of aglycemia on the CAP area. A, upper. Longitudinal section of adult mouse optic nerve exposed to 2 h of aglycemia demonstrating multiple focal swollen axonal regions. Selected swollen regions are indicated by (*). Scale bar 5 μm. A, lower. Model of a single INR with three swollen regions of variable diameter. (B) The control triple-peaked CAP and the attenuated CAP after the recovery phase following exposure to (i) 1 h or (ii) 2 h of aglycemia. Scale bars also apply to (ii). (C) Exposure to 1 or 2 h of aglycemia, indicated by horizontal bars, followed by a 1-h recovery in 10 mmol/L glucose aCSF, resulted in a delayed fall followed by incomplete recovery of the CAP, the degree of recovery decreasing with duration of exposure to aglycemia. (D) Simulation where the length of the swollen regions was 10 μm, and the diameter of the swollen regions increased from 1 μm (i) to 3 μm (ii), and 5 μm (iii) until conduction block occurred at 7.3 μm (iv).

The effects of metabolic disruption to central white matter axons resulting from aglycemia, anoxia, or ischemia include swollen sections of axon, which gives the axon a “string of pearls” appearance (Waxman et al. 1992; Garthwaite et al. 1999; Brown et al. 2001). An example of this effect in adult MON induced by 2 h of aglycemia is illustrated in Figure 6A, upper. This image shows a longitudinal section of MON with clear axon swellings evident throughout the tissue. The effect of multiple swellings within an individual INR was simulated with three swellings each 10 μm in length separated by myelinated regions of axons 12.5 μm in length, and of variable diameters (Fig. 7A lower). The effects of aglycemia on MON axon conduction were assessed by exposing MONs to either 1- or 2-h periods of aglycemia, followed by 1-h recovery in control aCSF containing 10 mmol/L glucose. In MONs exposed to 1 h of aglycemia, the CAP began to fall after 21.0 ± 3.7 min and fell rapidly to zero, where it remained for the duration of the aglycemic insult. On reintroduction of 10 mmol/L glucose aCSF, the CAP area slowly increased and recovered to 45.5 ± 18.8% by the end of the 1-h recovery period (Fig. 7B and C, *n* = 6). In MONs exposed to 2 h of aglycemia, the CAP fell after 19.3 ± 3.4 min and recovered to 16.6 ± 7.7% of control (Fig. 7B and C, *n* = 5, *P* = 0.018, vs. 1 h aglycemia, Fig. 7C). We simulated the effect of multiple swellings within a single INR (between nodes 10 and 11) on conduction velocity; the location of action potential simulations was the same as those illustrated in Figure 5. The simulation modeled the swollen regions of axon as demyelinated, as would presumably occur as a result of the swelling, but the unaffected regions of axon were modeled as being myelinated. Increasing the diameter of the swellings (Fig. 7D) from 1 μm (i), 3 μm (ii), and 5 μm (iii) caused attenuation of the conduction velocity until conduction block occurred at about 7.3 μm (iv).

### Effect of increased axolemmal permeability on conduction velocity

**Figure 8:**
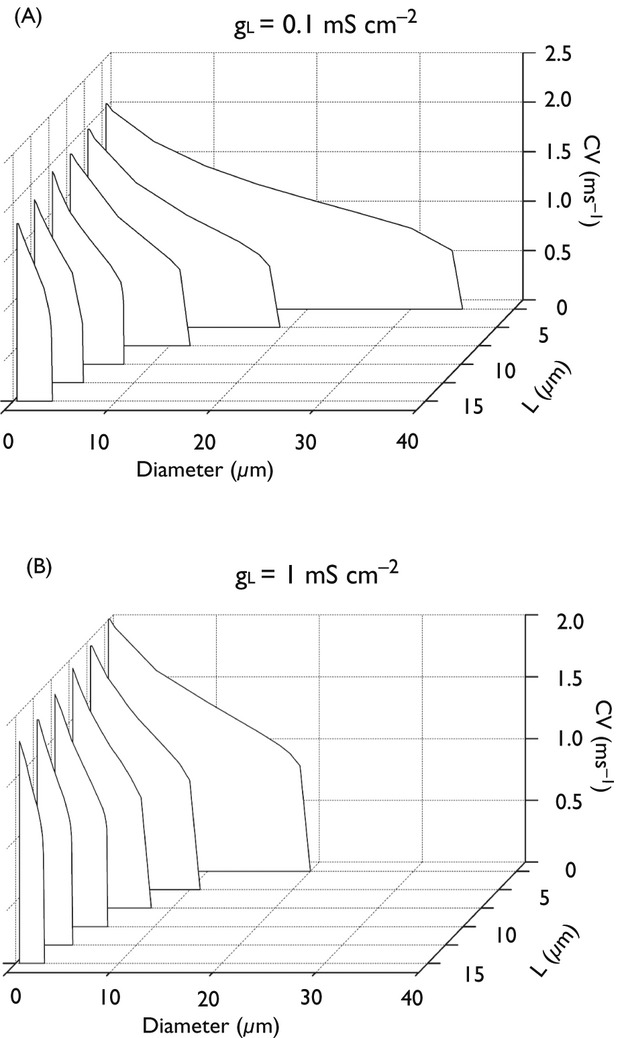
Effects of increased axolemmal permeability of conduction velocity. Simulations of three swellings in a single INR as illustrated in Figure 7A lower, and described in the text in the Results section demonstrated that increasing g_L_ from 0.1 mS cm^−2^ (A) to 1 mS cm^−2^ (B) decreases the swelling size at which conduction block occurs.

It is highly likely that swelling of the axon resulting from exposure to aglycemia would fracture the axolemma leading to an increase in permeability, that is, increased leak conductance across the axolemma, which we modeled by increasing the value of axolemmal g_L_. We increased g_L_ from the control value of 0.1 mS cm^−2^ (Fig. 8A) to 1 mS cm^−2^ (Fig. 8B) in simulations of three swellings ranging in length (L) from 2.5 to 15 μm within a single INR; our justification for the order of magnitude increase in g_L_ being the increased permeability of the axolemma to the tracer horseradish peroxidase clearly reflecting significant fracture of the axolemma (Pettus and Povlishock 1996). For the control value of g_L_ = 0.1 mS cm^−2^, conduction was maintained for swellings of lengths between 2.5 and 7.5 μm, with diameters greater than 10 μm. However, at swelling lengths of greater than 10 μm and diameters of less than 10 μm conduction failed. Qualitatively similar results were found with increased g_L_ of 1 S cm^−2^, but the dimensions at which conduction block occurred was much smaller within the dimensions of those measured experimentally. In MONs exposed to aglycemia, the dimensions of swellings can reach up to about 10 μm in length and diameter; similarly, in Alzhemier's disease, the axon spheroids can reach up to 10 μm in diameter (Adalbert et al. 2009).

### The mechanism of conduction block

**Figure 9:**
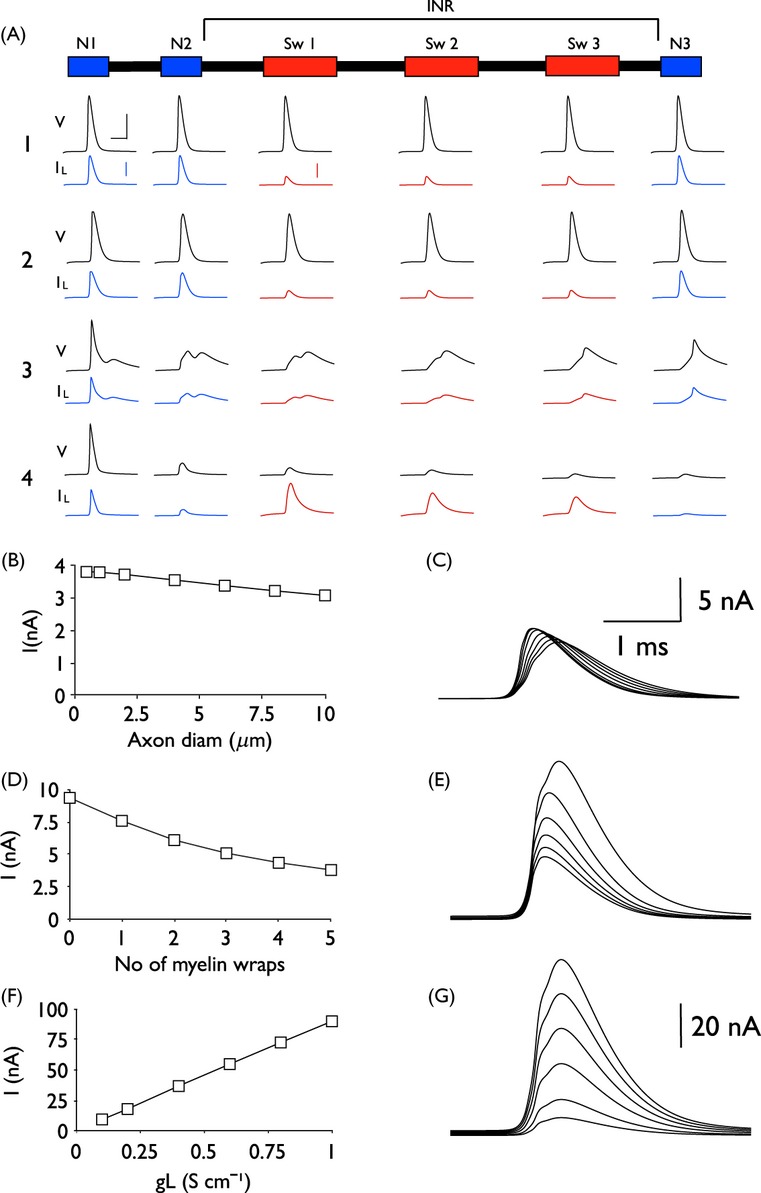
Transmembrane voltage and I_L_ resulting from the cumulative effects of swelling, demyelination, and increased axolemmal permeability. (A) Schematic of a region of axon with nodes and an INR containing three swollen regions of axon where voltage and current were recorded (voltage records are the black upper traces: scale bar 40 mV applies to all such traces. Horizontal scale bar is 1 ms and applies to all traces in A. Lower blues traces are nodal I_L_, and the blue scale bar is 5 nA which applies to all such traces. Lower red traces are I_L_ for regions Sw 1, Sw 2, and Sw 3 within the INR, and the red scale bar is 0.008 nA, which applies to all such traces). The axon regions were simulated under the following conditions, with the resulting voltage and current traces displayed underneath the corresponding region of the axon: Row 1. Control conditions where the INR is fully myelinated; thus, the regions denoted as Sw 1–3 have identical values to the control axon, for example, axon diameter = 0.48 μm, Cm = 0.0818 (μF cm^−2^), and g_L_ = 3.56 e-5 (mS cm^−2^). Row 2. The diameter of the three INR swollen regions (Sw 1–3) was increased to 10 μm; but otherwise the values of regions Sw 1–3 are the same as for Row 1. Row 3. The swollen region described in 2 was demyelinated; Row 4. The three swollen regions (Sw 1–3) were demyelinated and g_L_ increased from 0.1 mS cm^−2^ to 1 mS cm^−2^, but otherwise the values for Sw 1–3 are the same as for Row 3. The increased current at each of the three swollen regions and the decreased voltage response at N3 offer an explanation as to the mechanism of action potential failure. (B and C) Increasing Sw 1–3 diameters from 0.48 μm to 10 μm causes a slight decrease in the amplitude of I_L_. In C, the largest amplitude of I_L_ corresponds to the control axon diameter of 0.48 μm. Scale bar is 5 nA and 1 ms E. (D and E). Decreasing the number of myelin wraps around Sw 1–3 causes an increase in the amplitude of the transmembrane current. In E, the largest amplitude of I_L_ corresponds to 0 wraps of myelin. (F and G) Increasing the value of g_L_ at Sw 1–3 causes a linear increase in amplitude of the transmembrane current, which is about 10-fold the magnitude of the effect of demyelination. In G, the largest amplitude I_L_ corresponds to g_L_ of 1 S cm^−2^.

In an attempt to elucidate the mechanism(s) of conduction block resulting from focal axon swellings, we observed the magnitude of the transmembrane voltage and I_L_ at key regions of an axon, where the INR containing the swollen regions (Sw 1, Sw 2, and Sw 3) was abutted by nodes N2 and N3 (Fig. 9A, upper). The locations of the swollen regions within the INR are the same as those described in Figure 7A, lower. Under control conditions, where the swollen regions were myelinated, the transmembrane voltage and I_L_ at N1, N2, and N3 were identical, with the voltage at Sw 3 decreasing imperceptibly compared to Sw 1 (Fig. 9A, Row 1), due to the large value of λ. I_L_ at Sw 1–3 was much smaller than at the nodes due to the disparity in magnitude of g_L_ at the nodes (80 mS cm^−2^) compared to the INR (35.6 μS cm^−2^) (Table 1). Introducing swollen regions to the INR, by increasing the diameters of regions Sw 1–3 to 10 μm, had a minimal effect on both the transmembrane voltage and I_L_ compared to control (Fig. 9A Row 2). Modeling demyelination of the swollen regions resulted in an altered profile, with N2 I_L_ decreasing in amplitude, whereas I_L_ at Sw 1–3 increased in both amplitude and duration. The resulting voltage at N3 is attenuated and slower rising than control (Fig. 9A Row 3), but is of sufficient magnitude to promote distal action potential propagation (not shown). Imposing an increased g_L_ of 1 mS cm^−2^ in conjunction with demyelination of the swollen regions caused conduction block (minimal voltage change at N3) due to large I_L_ at (particularly) Sw1, Sw 2, and Sw 3 (Fig. 9A, Row 4). Comparison of I_L_ at Sw 1 reveals the cumulative effects of swelling, demyelination, and increased g_L_. Increasing axon diameter at Sw 1 from the control value of 0.48–10 μm caused a small decrease in I_L_ from 3.8 to 3.08 nA (Fig. 9B and C), while demyelinating the region caused an increase in I_L_ of 3.8 to 9.35 nA (Fig. 9D and E). However, the greatest effect on I_L_ occurred when g_L_ of regions Sw 1–3 was increased from 0.1 to 1 mS cm^−2^ (increasing g_L_ was carried out in conjunction with demyelination, as it is likely that demyelination will precede increased axolemmal permeability), with I_L_ increasing from 9.35 to 90.1 nA (Fig. 9F and G). The shunting of such large transmembrane currents via this increased axolemmal permeability results in insufficient current reaching N3 to depolarize the nodal membrane to threshold for action potential initiation, and thus conduction fails distal to this INR.

## Discussion

### The model

The model we developed of the myelinated axon is, in principle, similar to those described by others (Moore et al. 1978; Richardson et al. 2000; Devaux and Gow 2008; Bakiri et al. 2011) with the key characteristics of the model as follows: from a single soma extends the axon comprising alternating myelinated INRs interrupted by much shorter unmyelinated nodes of Ranvier. The morphological basis for the model was obtained from data in a recent publication, in which the authors used imaging techniques to view Lucifer yellow filled oligodendrocytes in corpus callosum and cerebellum to estimate the length of the myelinating processes, and axonal dimensions were estimated by imaging axon-specific antibody-labeled images (Bakiri et al. 2011). The passive electrical properties of oligodendrocytes were calculated from the responses of voltage clamped cells to voltage steps from the holding potential. The geometrical parameters of the myelin sheath, comprising five wraps of myelin, measured in the corpus callosum were 79.1 μm in length and 0.48 μm in diameter, with the underlying axon 0.36 μm in diameter, whereas the cerebellar myelin, comprising 10 wraps, was 106.1 μm in length and 0.96 μm in diameter (Bakiri et al. 2011). We have chosen to use the corpus callosum axon as our representative small CNS axon due to the myelinated axon diameter being similar to the median diameter of adult murine optic nerve axons (Allen et al. 2006), our model of choice for the last decade. However, we carried out all simulations based on data from both corpus callosum and cerebellar axons and found qualitatively similar results. The g ratio for both corpus callosal and cerebellar fibers was 0.75, close to the simulated optimal value in several computed models (Smith and Koles 1970; Moore et al. 1978; Chomiak and Hu 2009) and close to the measured value in morphological studies of the MON (Allen et al. 2006). Although there have been many models of myelinated axons, too numerous to cite here, the majority are models of peripheral fibers with axons up to 10 μm in diameter ensheathed with up to 150 wraps of myelin. The model we produced, as it utilizes morphological data from central white matter axons, can be reasonably expected to more accurately represent the small CNS axons than the generalizations drawn from modeling of large axons. Indeed, the constant g ratio described for corpus callosum and cerebellar axons is satisfying as it conforms to both the theoretical predictions (Waxman and Bennett 1972), and morphological measurement from axons in the optic nerve (Allen et al. 2006), of the smallest myelinated CNS axons of 0.2 μm diameter.

### Voltage-dependent conductances

In our model, in the absence of any data from optic nerve axons, we used historic data (Richardson et al. 2000). We justify this as a similar approach was taken by the authors who measured the morphological and electrical data of axons and oligodendrocytes upon which the model is based (Bakiri et al. 2011). In addition, the profile of our simulated action potential closely resembles that of an intracellularly recorded action potential from an optic nerve axon (Gordon et al. 1988), with a key feature being the lack of an after hyperpolarization. The voltage-dependent conductances are placed at the nodes, but are absent from the axon membrane in the INR. The model was populated with a fast I_Na_, a slow I_K_, and a persistent I_NaP_. The fast I_Na_, as encoded by Nav1.2, and the I_NaP_, as encoded by Nav1.6, are expressed at nodes of Ranvier in myelinated axons (Caldwell et al. 2000; Boiko et al. 2001; Waxman et al. 2004). Although this model conforms to previous historical models, accumulating evidence suggests that I_K_ channels are not only present at the nodes but also are present on the axolemma underlying the myelin, thus, any K^+^ efflux into the periaxonal space is shielded from the extracellular space by tight junctions (Devaux and Gow 2008). It has been proposed that, in the classical Hodgkin Huxley style, delayed rectifier channels play no part in the repolarizing phase of the action potential (Ritchie 1995), key arguments being the lack of a hyperpolarizing after potential (Kocsis and Waxman 1980), K^+^ accumulation in the periaxonal space following axon stimulation (Rasband 2004; Bellinger et al. 2008), and the contrasting effects of the specific Kv1.1 inhibitor DTX on control and claudin 11 null mice (Devaux and Gow 2008). The relatively large nodal g_L_ (80 mS cm^−2^) coupled with the fast time constant (τh) for inactivation of I_Na_ would result in rapid repolarization of the membrane potential in the absence of I_K_ (Ritchie 1995; Koch 1999). We modeled the lack of nodal I_K_ by removing the slow I_K_ from the model, and found the effect on conduction velocity and action potential profile to be negligible; conduction velocity decreased to 98% of control and the action potential profile was unchanged (data not shown). Although not included in our model for the sake of clarity, there is good evidence that a pathway connecting the periaxonal space and the extracellular space exists (Rosenbluth 2009; Mierzwa et al. 2010), the high resistance of which is maintained by claudin 11. In claudin 11 null mice, the conduction velocity is significantly slowed, suggesting that claudin 11 prevents current shunting via myelin and maintains the low capacitance of the myelinated INR (Devaux and Gow 2008; Gow and Devaux 2008).

### Demyelination and remyelination

Given the small diameter of the axon we modeled (0.36 μm), the number of myelin wraps was correspondingly low. These axons are at the lower end of size spectrum in the CNS, and would thus be expected to conduct comparatively slowly. We computed a control conduction velocity of 2.31 msec^−1^, comparable with a similar model based on the same morphological and electrical parameters (Bakiri et al. 2011). The effects of demyelination on conduction velocity were broadly as expected. Decreasing the degree, that is, the number of myelin wraps, caused a nonlinear incremental attenuation of conduction velocity as expected given the nonlinear relationship between INR capacitance relative to the number of myelin wraps (see eq. 2). Simulating complete demyelination at only one INR decreased conduction velocity from 2.31 to 1.59 msec^−1^, but did not block conduction, and such persistence of conduction through demyelinated axons has also been reported in previous models of demyelination (Waxman and Brill 1978). Simulating demyelination in multiple contiguous INRs resulted in a more profound attenuation of conduction velocity. Thus, in one INR reducing the number of myelin wraps from five to zero reduced conduction velocity to 1.59 msec^−1^. A similar decrease in conduction velocity (1.56 msec^−1^) could also be achieved by reducing the number of myelin wraps to three in six contiguous INRs. This key point that focal demyelination does not have as great an effect on conduction velocity as more general dys- or demyelination may explain the more profound functional consequences that occur in diseases producing more generalized dysmyelination, such as Pelizaeus Merzbacker disease (PMD; a leukodystrophy) or periventricular leukomalacia.

### Na^+^ channel expression

The presence of myelin on axons promotes rapid conduction, but its presence is accompanied by a few key constraints. In order for myelin to effectively promote conduction, the distance between the nodes must be long, as the capacitative charging of the nodal membrane that preempts ion channel activation delays the propagation of the action potential. However, the INR must be sufficiently short, such that the axoplasmic current does not dissipate across the axolemma before charging the next node. We have demonstrated a safety factor for g_Na_ of almost 4 (i.e., the control value of g_Na_ [3 S cm^−2^] divided by 0.81 S cm^−2^, the value of g_Na_ where conduction is blocked [Fig. 3B]), where a sufficient density of nodal Na^+^ channels must be present in order for the membrane potential to reach threshold rapidly, but not so many as to waste energy required for both the transport and maintenance of the channels distant from the cell body site of manufacture (Zhang et al. 2012), and for the equilibration of transmembrane ion gradients that is an inevitable consequence of action potential firing.

Demyelination results in an imbalance between channel expression and the resistive and capacitative properties of the denuded axons, an impedance mismatch, whose effect is to allow current dissipation across the previously myelinated axolemma. Demyelinated axons can express Na^+^ channels in the absence of remyelination with the Na^+^ channels expressed on the bare axon (Craner et al. 2004). Our simulations indicate that although redistribution of nodal channels onto axons at a variety of densities throughout the axons increased the conduction velocity relative to total loss of all expression of channels abutting the demyelinated INR, the values attained were always lower than control conduction velocity. An even distribution of g_Na_ and g_NaP_ across the nodes and denuded axons (Fig. 3D h) results in lower conduction velocity due to lower density of nodal g_Na_ and g_NaP_ compared to other simulation scenarios (Fig. 3D e,f,g). Matching control conduction velocity could only be achieved by simulating expression of additional Na^+^ channels. For example, at an even density of expression throughout the entire denuded INR of between 5% and 10% of control expression for g_Na_ and g_NaP_, respectively, was required, which taking into account the relative surface area of the axon compared to the node, a factor of 79.1, is many fold greater than control expression of the channels at the nodes. Such an increase in post demyelinated Na^+^ channel expression (Nav1.2 and Nav1.6) would require trafficking of ion channel proteins from the distant soma, but this phenomenon has been reported (Craner et al. 2004), although whereas the role of Nav1.2 channels is to promote axon conduction, the axonal expression of the persistent Nav1.6 is thought to lead to toxic Ca^2+^ overload and axon death.

### Repetitive firing

The model can generate repetitive firing activity in response to either current injection or decreasing nodal g_L_. Whereas the Hodgkin Huxley model accurately described the changes in membrane permeability underlying an individual action potential (Hodgkin and Huxley 1952), the model is less successful in describing repetitive firing activity (Koch 1999) due to the lack of a rapidly inactivating I_A_ type current. The limited range of stimulus current that induces repetitive firing in our model, the limited range of frequencies at which the model fires, and the nonzero frequency at which repetitive firing commences (Hopf bifurcation) are all reminiscent of Hodgkin Huxley–type models lacking K^+^ conductances that limit firing frequencies (Koch 1999).

Reduction in the nodal g_L_ current induces repetitive firing in the absence of external stimulus. The mechanism behind this phenomenon has been described previously and is dependent upon the ratio of g_Na_/g_L_; the larger the ratio the more unstable the system (Coggan et al. 2010). An interesting feature of the system of relevance to our model is the requirement of a persistent Na^+^ current to sustain the repetitive firing activity (Fig. 4C v).

### Swollen axons

The effect of changes in axon diameter on action potential conduction was first modeled almost 40 years ago, and showed that propagation of action potentials is very sensitive to the changes in axon dimensions (Goldstein and Rall 1974). Briefly, the results showed that action potentials approaching a narrowing region of axon increased velocity (and height), but thereafter the velocity decreased. Conversely, action potentials approaching a region of increased cylinder diameter decrease in velocity, but propagation sped up past the step change in diameter. However, increasing the diameter by more than a factor of 3.5 resulted in a failure of conduction (Goldstein and Rall 1974), whereas in our model conduction failed when the ratio of the large versus small axon diameter exceeded 9 (data not shown). We modeled the effect of an abrupt change in axon diameter from 0.48 μm to 0.6 μm with appropriate changes in C_m_ and g_L_ (data not shown). The key changes were decreases in C_m_ and g_L_ amplitude in the larger axon associated with the action potential, and an increase in conduction velocity as expected. In our simulations, the length of the swollen regions are relatively small compared to the lengths of the INRs and the length of the axon over which simulated action potentials were measured, thus any increases in conduction velocity due to the increased diameter of the swellings would be minimal relative to the attenuating effects of demyelination and increased axolemmal permeability on conduction velocity.

The effects of altering the basal axon diameter on swelling-induced conduction block demonstrated that in larger axons larger sized swellings were required to induce conduction block, indicative of a threshold of the ratio between basal axon diameter and swelling dimensions that must be reached in order for conduction to be blocked. Similarly, conduction block can be overcome by marginally increasing the basal axon diameter (by as little as 0.01 μm) without altering the dimensions of the swellings.

The focal swellings that are the hallmark of *Plp1* and *Cnp1* mutant axonopathy are probably limited to a single swelling per INR and possibly even to a single swelling per axon, at least initially (Edgar et al. 2004). The swollen regions contain aggregates of axoplasmic organelles such as mitochondria and dense bodies, and are frequently located at the distal juxtaparanodal region in the *Plp1* knockout mouse. Paranodal swellings have also been reported by others (Nikic et al. 2011; Ip et al. 2012). Whereas in the *Plp1* mutant, where it has been shown that the myelin sheath is often both attenuated and retracted in regions of swelling, compromising its integrity, and thus its role as a high-resistance, low capacitance insulator (Edgar et al. 2004), in *Cnp1* mutants there is no focal demyelination accompanying axon swelling (Edgar et al. 2009). In our simulation, individual swellings of length 10 μm required dimensions greatly in excess of those observed experimentally to produce conduction block, and conduction velocity was barely attenuated at physiologically relevant dimensions. This is in agreement with our electrophysiological recordings of the *Cnp1* mutant where there was no obvious difference between the CAP profile recorded from WT or mutant mice.

Given the previous results that demonstrate conduction persists in totally demyelinated axons, and that increasing axon size of demyelinated axons leads to increased conduction velocity (Nicholls et al. 2001), the ability of axons with multiple INRs each containing a single swelling to maintain conduction may not be surprising. However, as a result of metabolic perturbations such as aglycemia and/or anoxia, axonopathy results in multiple axon swellings within a single INR (Waxman et al. 1992; Garthwaite et al. 1999; Brown et al. 2001). These multiple regions would put more of a strain on the ability to conduct, as they would most likely be accompanied by demyelination and increased leak current across the axolemma (Pettus and Povlishock 1996; Povlishock and Pettus 1996; Fitzpatrick et al. 1998; Choo et al. 2008). In our model, with three evenly spaced swellings of length 10 μm, present in a single INR, the effect on conduction velocity was much greater than a single equivalent swelling of comparable dimensions. It is important to note that EM images show swellings with dimensions in the range simulated. These simulations were run with no overlying myelin, that is, g_L_ was 0.1 mS cm^−2^. Factoring in the increased g_L_ across the axolemma resulting from swelling greatly decreased the diameter required to block conduction; at g_L_ = 1 mS cm^−2^ a diameter of just over 6 μm blocked conduction (Fig. 8B). Our electrophysiological recordings clearly demonstrate conduction block in MONs exposed to aglycemia, with increased duration of exposure to aglycemia resulting in an increased number of nonconducting axons (Fig. 7).

It is clear from Figure 9 that swellings of the axon alone had a minimal effect on conduction velocity, in concord with our electrophysiological recordings, which showed no significant difference in the CAP between WT and *Cnp1* mutant mice. Although demyelination of swollen regions did attenuate conduction velocity, the increased current shunting across the axolemma was only about double that of control. By far the greatest effect on I_L_ and conduction velocity was increased axolemmal permeability, which increased current shunting by an order of magnitude compared to control. This had a substantial effect on the ability of axons to propagate action potentials, particularly when there are multiple swollen regions within a single INR (Fig. 9A, 4) as the extent of current shunting across multiple such regions ensures that insufficient current reaches the distal node to drive the membrane potential toward threshold.

In summary, we developed a computer model, implemented via the NEURON simulation environment, of small CNS myelinated axons based on experimentally acquired morphological and electrical data from corpus callosum axons, with the intention of investigating the effects of axonal swelling on conduction velocity. Axon swellings can result from a variety of pathological conditions, which can be realized as single swellings, or multiple swellings in a single INR; such swellings are often accompanied by loss of overlying myelin and increased axolemma permeability. Our simulations demonstrated an attenuation of conduction velocity commensurate with increased dimensions of the swollen region, with increased axolemma current shunting acting in conjunction to block conduction. These results have implications for understanding neurological dysfunction as a result of metabolic insult where multiple swollen regions can be present within a single INR (Waxman et al. 1992; Garthwaite et al. 1999; Brown et al. 2001), and in neurodegenerative disease including myelin disorders in which axonal swellings often occur on otherwise intact axons (Edgar et al. 2004, 2010; Bridge et al. 2009; Mierzwa et al. 2010; Nikic et al. 2011).
